# Density Functional Theory Guide for an Allyl Monomer Polymerization Mechanism: Photoinduced Radical-Mediated [3 + 2] Cyclization

**DOI:** 10.1021/acsomega.1c00165

**Published:** 2021-06-08

**Authors:** Xiaotian Zhao, Wanqiu Huang, Shibo Lin, Xi Chen, Xirui Guo, Dehong Zou, Guodong Ye

**Affiliations:** † Chengdu Second Peoples Hospital, Chengdu 610017, P.R. China; ‡ The Fifth Affiliated Hospital of Guangzhou Medical University, Guangzhou 510799, P.R. China; § Guangdong Pharmaceutical University, Guangzhou 510006, P.R. China

## Abstract

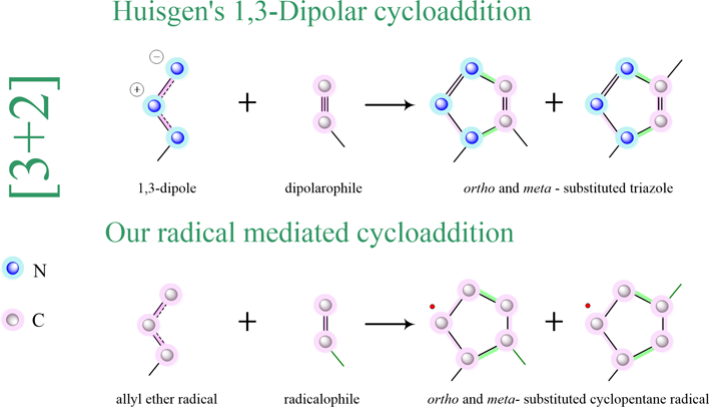

Polymerization of allyl ether monomers has previously been considered a free-radical addition polymerization mechanism, but it is difficult to achieve because of the high electron density of their double bond. To interpret the mechanism of photopolymerization, we therefore proposed a radical-mediated cyclization (RMC) reaction, which has been validated by results from quantum chemistry calculations and real-time infrared observation. Our RMC reaction begins with the radical abstracting one allylic hydrogen atom from the methylene group of allyl ether to generate an allyl ether radical with a delocalized π_3_^3^ bond. Then, the radical reacts with the double bond of a second allyl ether molecule to form a five-membered cyclopentane-like ring (CP) radical. The CP radical abstracts a hydrogen atom from a third ether molecule. At last, a new allyl ether radical is generated and the next circulation as chain propagation begins. The distortion/interaction model was employed to explore the transient state of reaction, and real-time infrared was chosen to clarify the RMC reaction mechanism initiated by different photoinitiators. These results demonstrated that the RMC mechanism can give new insights into these fundamental processes.

## 1 Introduction

In recent years, there has been increased interest in allyl ether monomers^1^ because of their exceptional physical and electrical properties. They have found extensive use in many applications^2^ such as fibers, films, coatings, optical glasses, etc. Polymerization of allyl monomers is commonly thought to proceed *via* a free-radical addition (FRA) mechanism.^3,4^ According to this model, radicals decomposed from thermolysis/photolysis of initiators added to the double bonds of monomers to yield primary radicals. Successive addition of more allyl ether (AE) molecules to these primary radicals produces macromolecular products in the propagation step until the additions end in the termination step. In theory, AE molecules are not suitable for FRA polymerization because of their high double bond electron density, which is caused by the adjacent electron-donating methylene group. AE would be expected to polymerize slowly, producing oligomeric products, because of degradative chain transfer to the monomer instead of effective addition.^5,6^

Another expected issue was radical displacement reactions.^7^ Bulter and Angelo^8^ asserted that the most important chain propagation pathway in diene polymerization is cyclization with cyclolinear polymer products. However, in our experience, polymeric products with high molecular weights were also obtained easily.^9,10^ A noticeable monomer, sucrose allyl ether (SAE), was polymerized at room temperature using ultraviolet light, and the product (poly-SAE) was successfully applied in minimally invasive surgery^9^ as a hemostatic material.

**Scheme 1:**
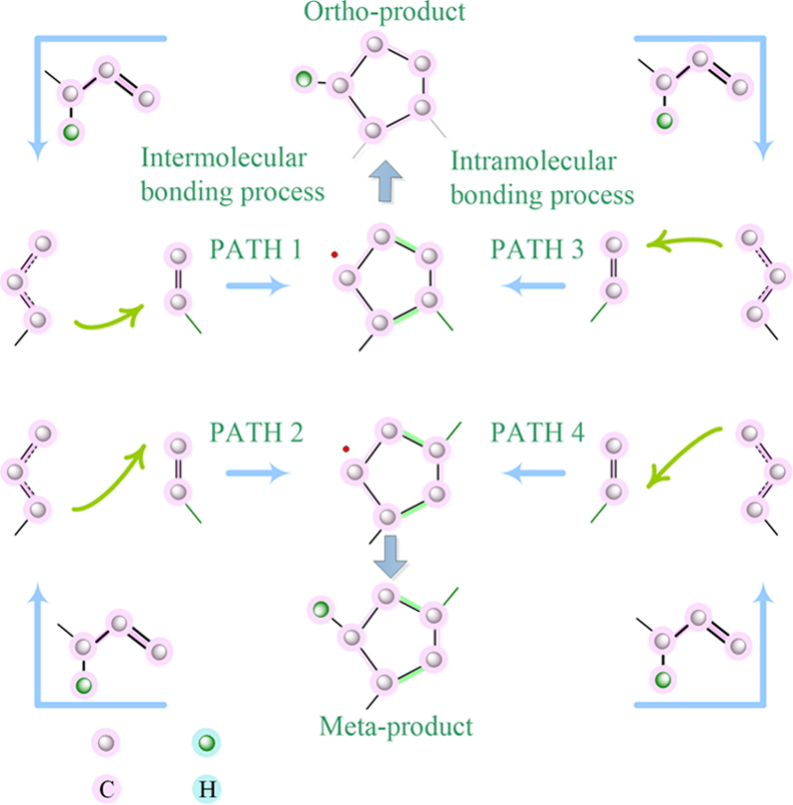
[3 + 2] Radical-Mediated Cyclization in Four Paths

In recent years, mostly new mechanisms were reported^11,12^ in many fields. In this study, we developed a mechanism called radical-mediated cyclization (RMC) to explain polymerization. The procedure and mechanism for this strategy are shown in Scheme 1. Our method promises to be useful in organic synthesis for obtaining [3 + 2] cyclopentane-like five-membered compounds and act as a new linkage route for various polymer architectures^13,14^ like the Huisgen 1,3-dipolar reaction.^15,16^ Generally, the π_3_^3^ allyl ether radical will be more important in cyclization reactions, as with the carbene or TEMPO^17^ nitroxyl radical.

In this work, first, in the Computational Methods section, we explored the hydrogen abstraction (HAT) process induced by photoinitiators and thermal initiators. We also investigated the bond dissociation energy (BDE) and electrostatic potential (ESP)^18^ of different allyl ether monomers. Then, we studied the mechanistic details of RMC. Finally, the transition state kinetic parameters of product radicals were also discussed to clarify the completed cyclization reaction process in the final stage, including the conformation of the product radical of the transient state (TS) and thermodynamic parameters (such as the free energy changes) of the reaction in combination with kinetic descriptors (such as rate constants). In the Experimental Section, poly-SAE was successfully synthesized and we also investigated the polymerizing kinetics of SAE initiated with different series of photoinitiators based on the time-resolved IR spectra in the search for experimental proof of our theory.

## 2 Results and Discussion

In Scheme 1, conceivable modes for the cyclization reaction to the respective in four paths are shown. However, the products are *meta-* and *ortho-*products.

### 2.1 Initiation Behavior

The RMC model also explains the different reactivities of thermal initiators AIBN and BPO when used with SAE. HMPP, AIBN, and BPO generate a benzoyl radical, 2-cyano-2-propyl radical, and benzoate radical, respectively. The photolysis reaction of cleavage and hydrogen abstraction photoinitators is shown in the Supporting Information. Before the RMC reaction, in the hydrogen abstraction, the benzoyl radical or 2-cyano-2-propyl radical abstracted one allylic hydrogen atom from the methylene group in AME. However, the benzoate radical due to its stable conjugate structure reacted with AME difficultly.

However, since the phenyl radical is the product of benzoate radical decarboxylation, the phenyl radical was selected for HAT. For comparison, the benzoyl, 2-cyano-2-propyl, and benzoate radicals, respectively, were added onto the double bonds of AME as the first step of the FRA reaction. For HMPP, when the benzoyl radical reacts with AME by photopolymerization, as shown in Table 1, the two *E*_a_ values for HAT and FRA are close. However, the Δ*G* (−12.22 kcal·mol^–1^) in HAT is much more negative than that in FRA (−1.31 kcal·mol^–1^), providing a huge reaction driving force and indicating that the benzoyl radical tends to react with AME *via* HAT. For AIBN, when the 2-cyano-2-propyl radical reacts with AME by thermopolymerization, the two *E*_a_ values of CP reacted with AME were almost equal, but Δ*G* (10.13 kcal·mol^–1^) in FRA is positive, indicating that the FRA reaction is not spontaneous. With AIBN initiation, HAT is spontaneous (Δ*G* = −3.40 kcal·mol^–1^). However, its driving force is so small that the reaction would not proceed to completion. This is the reason AIBN cannot initiate the polymerization of SAE. For BPO, the *E*_a_ and Δ*G* of the phenyl radical reaction with AME are far more suitable than those of the benzoate radical. This suggests that HAT is better than FRA if enough BPO can convert into the phenyl radical, which explains our observation that allyl ether began to ploymerize only on addition in high concentrations of BPO. However, we do not know how much time was required for decarboxylation of the benzoate radical. This means that HAT might not occur without obvious decarboxylation. As we predicted, FRA, including the degradative chain transfer model, is not suitable for multi-allyl ether monomers. The HAT reaction proceeds more easily than FRA, proving the correctness of HAT as the first stage of the RMC reaction.

**Table 1:** Comparison of Thermodynamic Properties between Hydrogen Atom Transfer (HAT) and Free-Radical Addition (FRA) Reactions when Fragment Radicals of Initiators Reacted with Allyl Methyl Ether (AME) as a Model Compound of SAE^a^

initiator	radical	reaction type	Δ*H* (kcal·mol^–1^)	Δ*G* (kcal·mol^–1^)	*E*_a_ (kcal·mol^–1^)
HMPP	benzoyl	HAT	–12.00	–12.22	19.38
	benzoyl	FRA	–12.87	–1.31	18.97
AIBN	2-cyano-2-propyl	HAT	–4.82	–3.40	25.54
	2-cyano-2-propyl	FRA	–2.84	10.13	25.48
BPO	phenyl	HAT	–33.99	–34.35	10.28
	benzoate	FRA	–16.56	–4.89	18.17

a Δ*G*: Gibbs’ free energy change/reaction drive force; *E*_a_: activation energy; Δ*H*: enthalpy change; HAT: hydrogen atom transfer reaction; FRA: free-radical addition reaction; functional: B3LYP, basis set: 6-311++G(d,p).

Condensation polymerization can be summarized as HAT followed by cyclization. We call the mechanism RMC because the radical-containing π_3_^3^ occurs as an important intermediate. Notice that contrary to Barnett and Butler’s assertion,^19^ the important characteristic of our cyclization is the product containing a cyclopentane-like five-membered ring. The present model accounts well for the known features of the low polymerization rate because propagation occurs when the secondary/tertiary carbon radical of a cyclopentane-like ring abstracts a new allylic hydrogen atom from the methylene group of allyl ether. From our experience using photoinitiators, we believe that the π_3_^3^ radical can be obtained not only from free radicals like benzoyl but also from triplet state compounds such as ITX. The cyclization reaction does not resemble the Diels–Alder reaction because the [3 + 2] ring formation proceeds *via* a stepwise or asynchronous mechanism. However, this cyclization seems more plausible than the radical displacement reaction suggested by Gaylord^20^ to explain multi-membered ring formation during polymerization.

### 2.2 Allyl Monomer Descriptors

**Figure 1:**
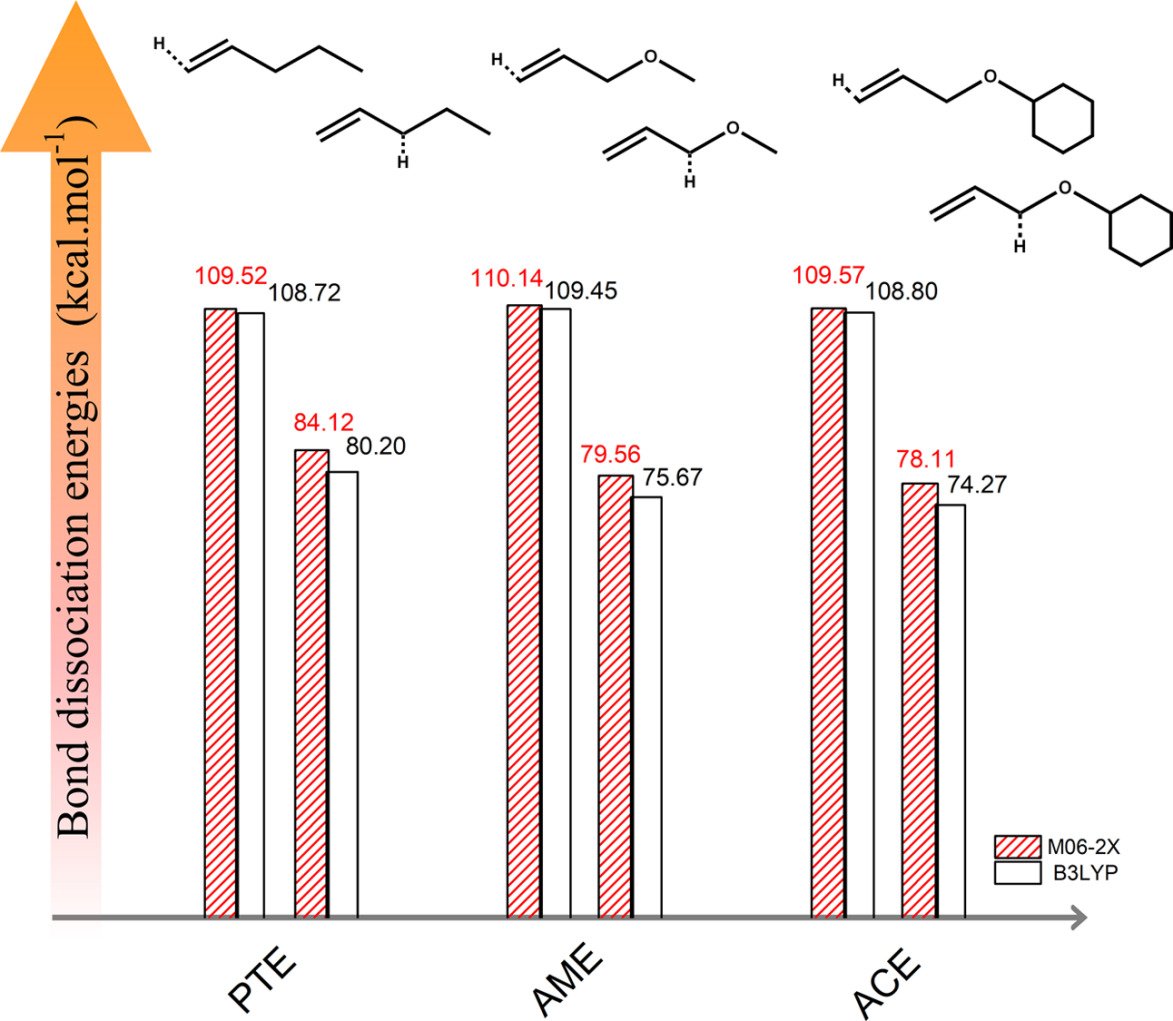
Bond dissociation energies of C–H bonds in different donors.

Since HAT is the first step, the BDE of C–H bonds in donors is the key to hydrogen transfer.^21^ As can be seen from the data in Figure 1, PTE, AME, and ACE are all allyl monomers only with a different substituted group. According to Figure 1, we found that the BDEs for the methylene group of the three monomers are in general lower. For example, the BDE value of H in the terminal group of AME is higher (approximately 30 kcal·mol^–1^) than that of the methylene group. Furthermore, the BDE value of the allyloxy donor monomers (AME and ACE) for the methylene group is similar (about 75 kcal·mol^–1^) at the B3LYP/def2tzvp level, and it is lower than PTE. We considered two effects to be responsible for the differences of the absolute BDE data of the three monomers: one is the oxygen atom and the other owing to a different substituted group. From the molecular orbital approach, the population analysis by using either the natural bond orbital or the Mulliken scheme, the interacting orbitals appear to be not the same in these monomers. In order to study the donor’s electronic character, we portray the ESP picture below.

**Figure 2:**
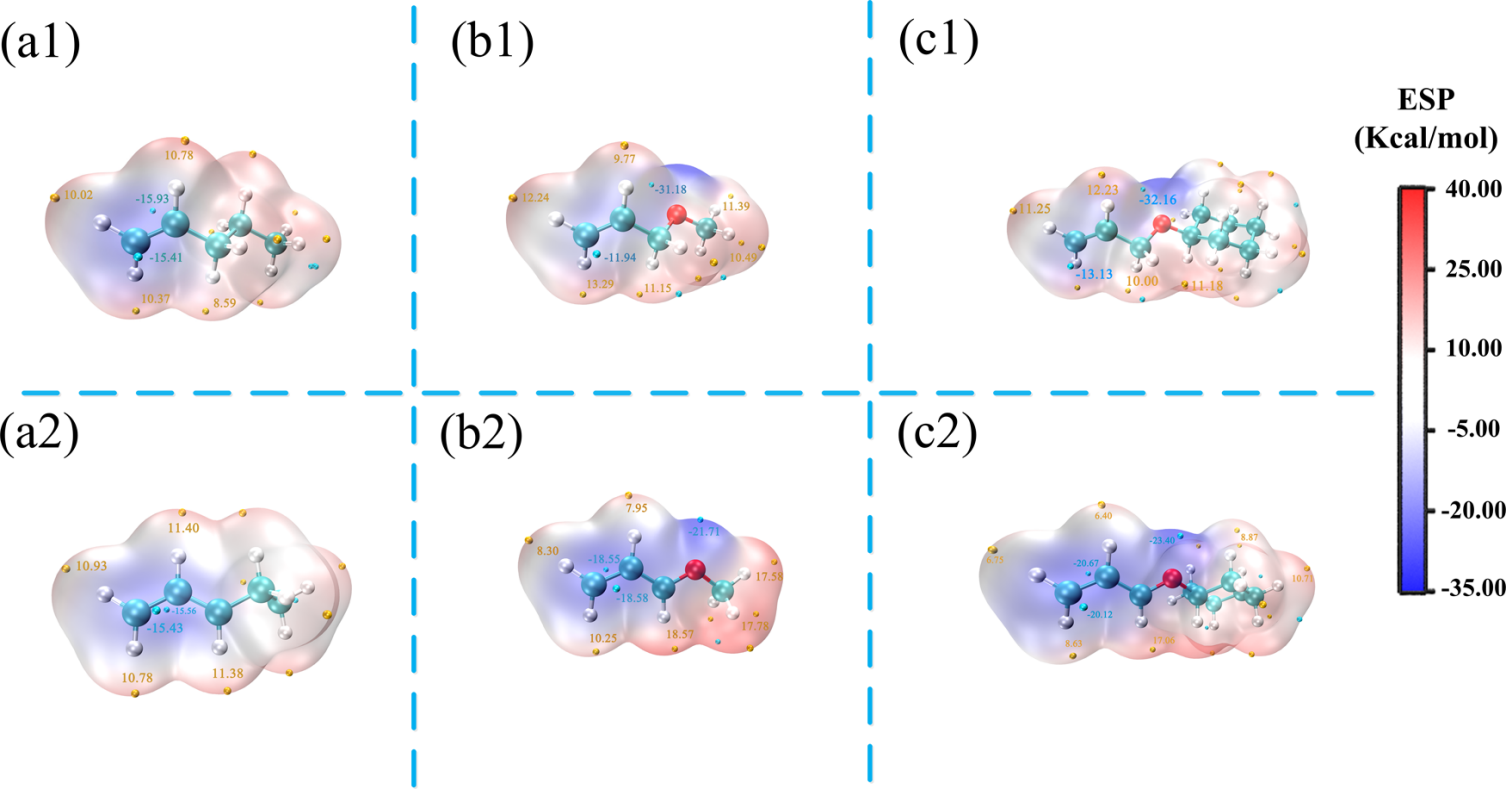
ESP mapped molecular vdW surface of the three allyl monomers: (a1) PTE, (a2) PTE radical, (b1) AME, (b2) AME radical, (c1) ACE, and (c2) ACE radical. Significant surface local minima and maxima of ESP are represented as gold and cyan spheres and labeled by yellow and blue texts, respectively.

ESP on the molecular surface is critical for understanding and predicting intermolecular interaction.^22^ In-depth investigation of ESP of allyl monomers must be helpful for exploring reactivity. The ESP mapped molecular vdW surface of allyl monomers is shown in Figure 2. To further understand the characteristics of the allyl groups, we calculated the electric descriptors about allyloxy compounds AME and ACE of its radical structures. The site possessing more negative ESP has a stronger ability to attract electrophiles and thus is more possibly to be the reactive site. The surface area in each ESP range is plotted in Figure 2. From Figure 2c2, it can be seen that in the allyloxy region in the ACE radical, the surface minima of ESP are present between -O-CH_2_-CH=CH_2_ carbon atoms, and the vdW surface has a large negative value of ESP around −21 kcal·mol^–1^ (i.e., −23.40 kcal·mol^–1^); they are attributed to the surface close to the oxygen atom and the different substituent groups. According to the surface minima area of the radical section (bottom of Figure 2), the negative value of ESP tends to have more equal distributions around allyl groups. The two sections of (a1) and (a2) show that the PTE molecular vdW surface has the same ESP value (i.e., within −16 to 12 kcal·mol^–1^); these regions belong to the double bond unit. The substituent group and oxygen in allyl units are the main contributors to the surface area having an ESP value lower at the -O-CH_2_-CH=CH_2_ groups (i.e., −18.58 kcal·mol^–1^ of the AME radical and −20.12 kcal·mol^–1^ of the ACE radical). This observation well explained the more electrophilic reactivity of allyl radical monomers. Our interest was focused on the reaction path of both donor- and acceptor-type monomer functions as well as on the [3 + 2] cyclization mechanism of the obtained polymers.

### 2.3 RMC Reaction

The reactions, in combination with the previously noted mechanism of cyclization, are briefly depicted in Scheme 1. The probable mechanism involves the photoinduced allyl monomers in the first step. Subsequent abstraction of hydrogen from a new allyl monomer promotes the formation of *ortho-* and *meta-* ring radicals. As an electron-donating group, allyl methylene exerts a powerful effect on the double bond. The double bond electron density of SAE or AME is higher than that of a normal olefin (e.g., ethene) and far higher than those of electron-deficient monomers such as acrylates, making polymerization difficult. Presumably, at best, a small amount of low-molecular-weight oligomer might be obtained from radiation-induced SAE polymerization using photoinitiators. For example, to initiate, vinyl ether requires iodonium or sulfonium salts, and the reaction proceeds *via* cationic polymerization. Our quantum chemistry calculation showed (Figure S1) that when a benzoyl radical from photocleavage of HMPP is added onto the double bond of AME, the addition rate constant (*k*) in the gas state is 4.6 × 10^–21^, one thousand times slower than *k* for addition to acrylate (2.2 × 10^–18^).^23^ However, in a previous study,^10^ we observed that SAE polymerization proceeded at a fairly rapid rate and formed a stable polymer, a phenomenon that is difficult to explain using the traditional FRA polymerization mechanism.

**Figure 3:**
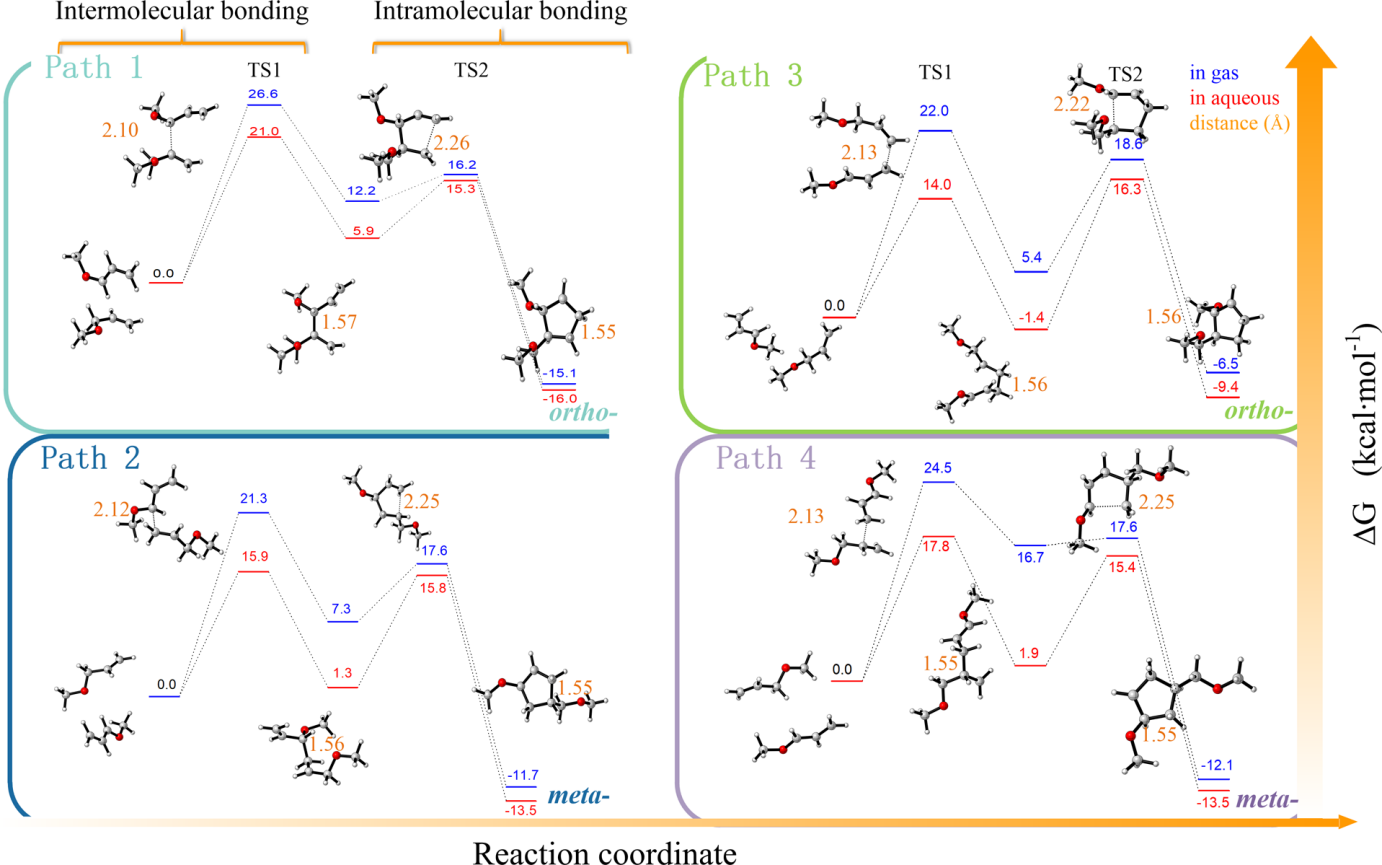
Gibbs free energy change (kcal·mol^–1^) potential map using allyl methyl ether (AME) as a model compound based on B3LYP/6-311++G(d,p) in the gas phase (blue color) and aqueous solution (red color) at 298.15 K. HAT: hydrogen atom transfer; RMC: radical-mediated cyclization.

Therefore, we proposed the hypothesis that in the first stage, the benzoyl radical abstracted one allylic hydrogen atom from the allyl ether methylene group in preference to addition onto the double bond and then generated an allyl ether radical with a three-electron three-center delocalized π_3_^3^ bond as the primary radical. A reason for this is the low BDE of the hydrogen donor methylene sp^3^ C–H bonds. In the second step, the dehydrogenated allyl ether radical reacted with a second allyl ether molecule (not radical) to form a five-membered cyclopentane-like ring radical with two substituted oxygen-containing groups. This cyclization reaction occurs between the electron-deficient delocalized three-center π_3_^3^ bond (-O-C·H-CH=CH_2_) and the electron-rich carbon–carbon double bond (-O-CH_2_-CH=CH_2_). In the final stage, the five-membered ring radical abstracts another allylic hydrogen atom from a third neutral allyl ether molecule and continues the next circulation process to form the cyclolinear matrix of cyclopentanes and sucrose residues. In this case, two allyl ether molecules are needed to form a ring. As shown in path 1 of Figure 3, the first reaction was an intermolecular bonding process, forming the first single bond between the -O-C·H-CH=CH_2_ carbon atom in the radical and the -O-CH_2_-CH=CH_2_ carbon atom in the monomer with *E*_a_ = 26.6 kcal·mol^–1^ at TS1 in the gas state (data in blue). The product was a quasi-cyclopentane radical intermediate with Δ*G* = 12.2 kcal·mol^–1^. The second process was an intramolecular bonding process, forming the second single bond between two terminal =CH_2_ carbon atoms with *E*_a_ = 16.2 kcal·mol^–1^ at TS2, producing an *ortho*-disubstituted five-membered ring radical with Δ*G* = −15.1 kcal·mol^–1^.

The length of the first single bond (shown in orange) changed from ca. 2.10 Å in TS1 to ca. 1.57 Å in the intermediate, continued to become ca. 1.56 Å in TS2, and finally approached ca. 1.54 Å. The length of the second single bond between two =CH_2_ changed along the reaction coordinate from ca. 2.26 Å in the intermediate to ca. 1.55 Å in the product, indicating that the two new bonds in the CP radical are very similar. Since the allyl ether monomer is an asymmetric alkene, it may have followed path 2, i.e., the first single bond formed between the −C·H- carbon atom in the radical and the terminal **-**O**-**CH_2_**-**CH=CH_2_ carbon atom in the monomer with *E*_a_ = 21.3 kcal·mol^–1^ at TS1 and Δ*G* = 7.3 kcal·mol^–1^ in the intermediate. The second single bond was generated from the terminal =CH_2_ carbon atom in the radical and the **-**O**-**CH_2_**-**CH=CH_2_ carbon atom in the monomer, with *E*_a_ = 17.6 kcal·mol^–1^ at TS2 and Δ*G* = −11.7 kcal·mol^–1^ in the *meta*-disubstituted CP radical. The bond length change is similar to that predicted by path 1. The energy changes in the aqueous phase resemble those in the gas phase (shown in red). More interesting is that the Δ*G* in water become 1.3 kcal·mol^–1^ at TS1, indicating that the reaction is a near equilibrium state, not nonspontaneous like that in the gas state.

In our case, photopolymerization using asymmetric alkenes would produce a mixture of the two (*ortho*- and *meta*-) regioisomers. However, the *E*_a_ in the gas phase of path 2 was ∼3 kcal·mol^–1^ lower than that of path 1, and this difference becomes larger (∼5 kcal·mol^–1^) in the aqueous phase. Similarly, comparing paths 3 and 4, the results were obtained that showed that the *meta*-product is stable. In conclusion, lower-energy path 2 might be the dominant process and the *meta*-disubstituted compound is the major product.

### 2.4 Reaction Parameters

In our hypothesis, the HAT reactions may occur with production in the final stage, and with *meta*- and *ortho*-products as donors, they can react with the third AME molecular quickly. We investigated the HAT reaction of the above two ring products. The thermodynamic properties including Δ*_r_G*, *E_a_*, and Δ*_r_H*, are summarized in Table 2.

**Table 2:** Thermodynamic Parameters of Hydrogen Abstraction Reactions in the Final Stage^a^

				*E*_d_ (kcal·mol^–1^)			
	Δ*_r_H* (kcal·mol^–1^)	Δ*_r_G* (kcal·mol^–1^)	*E*_a_ (kcal·mol^–1^)	total	donor	acceptor	*E*_i_(kcal·mol^–1^)
AME + *ortho-*	–18.72	–19.24	21.01	11.41	8.46	2.95	9.60
AME + *meta-*	–18.23	–18.99	21.81	12.31	8.31	4.00	9.50

a *Ortho-: ortho*-product radical; *meta-: meta-*product radical; Δ*_r_H*: enthalpy change; △*_r_G*: Gibbs’ free energy change/reaction drive force; *E*_a_: activation energy; *E*_d_: deformation energy; *E*_i_: interaction energy; functional: B3LYP, basis set: 6-311++G(d,p).

Applying distortion/interaction model^24,25^ methods, we anatomized the components of *E*_a_, which comprises interaction energy (*E*_i_) and deformation energy (*E*_d_) and is the difference between them. The *E*_d_ is caused from stretched bonds or changed angles. Calculations show that the *E*_a_ of *ortho*- is slightly lower than that of *meta*-, having values of 21.01 and 21.81 kcal·mol^–1^, respectively. At room temperature, this difference in the height of the barrier means that *ortho*- reactions are formed rapidly than *meta*-, as confirmed by the reaction rate constants shown subsequently. According to the Δ*_r_H* value in Table 2, it is similar to that for Δ*G*, indicating the TΔ*S* is nearly zero. The change in entropy is a measure of the degree of disorder in the TS, and the value of the change in entropy will decrease or become negative. Therefore, the hydrogen transfer reaction can be called an isoentropy reaction. Both of the reactions are exothermic, which represents a negative change in enthalpy. The value of Δ*_r_G* of *ortho*- was found to be −19.24 kcal·mol^–1^, which was similar with the *meta*- (−18.99 kcal·mol^–1^), and it indicates that a large driving force is still stored in the product.

The kinetic parameters including bond orders (*n_T_*) and Wigner tunneling factors κ_(*T*)_ were obtained from the results of optimization and are listed in Table 3. The *n_T_* was also calculated according to the bond energy–bond order model^26^ as expressed in eq 1. The κ_(*T*)_ is expressed by the following eq.2
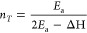
1
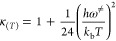
2where κ_(*T*)_ is the Wigner tunneling factor, ω^≠^ is the imaginary frequency of the transition state, *T* is the temperature, *k*_b_ and *h* are the Boltzmann constant and Planck constant, respectively, and *n_T_* is the criteria for “earliness” or “lateness” of the TS. The larger the value of *n*_T_, the later the TS would appear. From Table 3, it can be seen that the values of *n*_T_ were found to be equivalent or nearly so. This implies that there is no difference in the appearance of TS between *ortho-* reactions and *meta-* reactions. In addition, the ω^≠^ of *ortho-* is a little bit lower than that of *meta*-, so the corresponding κ_(*T*)_ become very lower. According to our observation, the κ_(*T*)_ value induced by radicals is usually over 3 and it has pronounced impact on the reactivity.^27,28^ The rate constant for *ortho-* (3.52 × 10^–22^ cm^3^·molecule^–1^·s^–1^) is about one order of magnitude higher than that for *meta-* (9.07 × 10^–23^ cm^3^·molecule^–1^·s^–1^) at the B3LYP functional, which also suggests that *ortho-* is more efficient in the hydrogen abstraction reaction.

**Table 3:** Imaginary Frequencies, Tunneling Factors, Rate Constants, and Bond Orders of the Six Reactions in the Final Stage^a^

	ω^≠^	κ_(*T*)_	*k* (cm^3^·molecule^–1^·s^–1^)	*n_T_*
AME + *ortho-*	–1613.17	3.53	3.52 × 10^–22^	0.27
AME + *meta-*	–1601.95	3.49	9.07 × 10^–23^	0.35

a κ_(*T*)_: tunneling coefficients; *k*: rate coefficients; *n_T_*: bond order; ω^≠^: imaginary frequency; functional: B3LYP, basis set: 6-311++G(d,p).

### 2.5 Infrared Spectra

**Figure 4:**
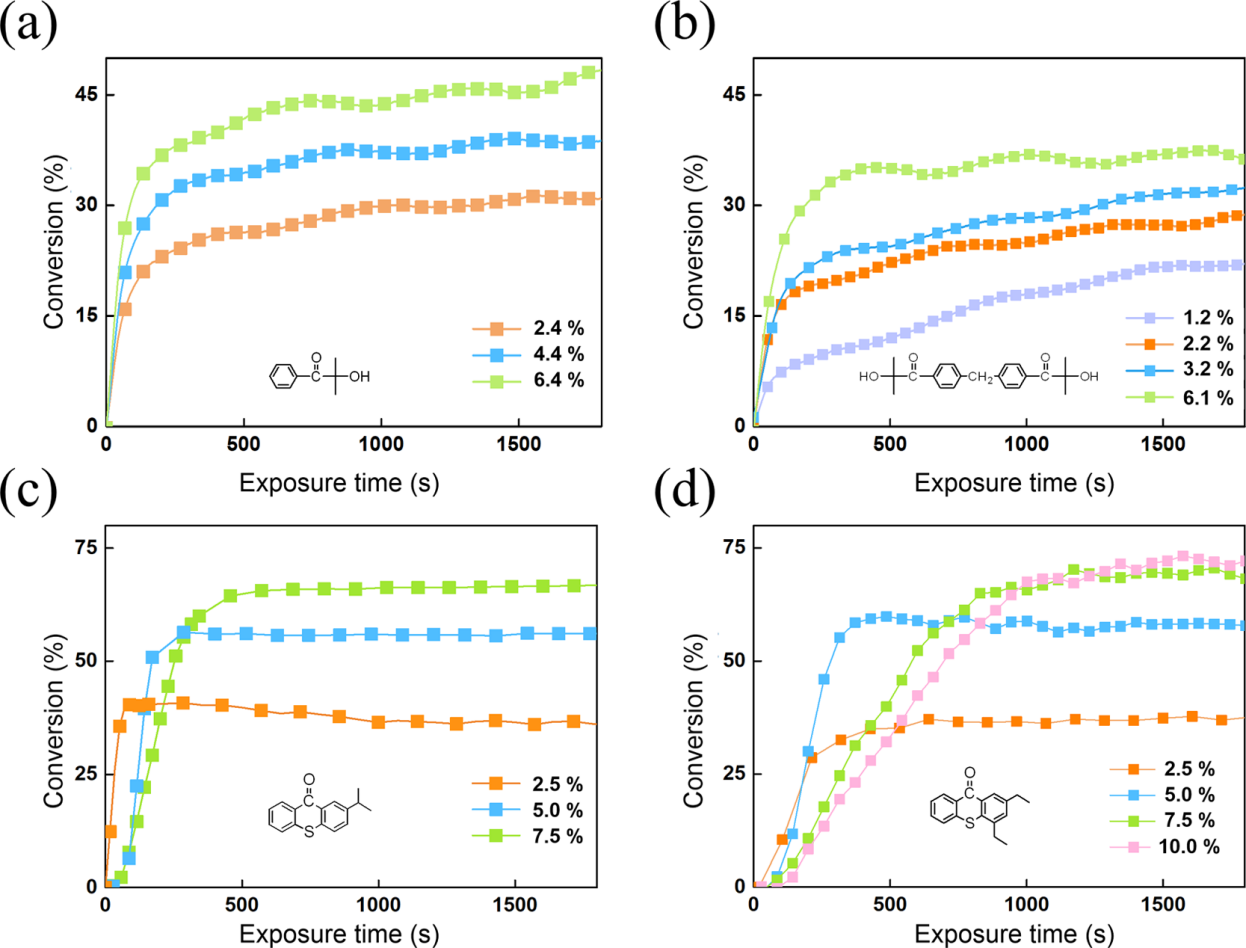
Photopolymerization profiles of the SAE monomer irradiated at 28 mW·cm^–2^ using different photoinitiators: (a) HMPP; (b) 127; (c) ITX; and (d) DETX.

We used real-time Fourier transform infrared (RT-IR) to test our hypothesis. HMPP and Irgacure 127, two photoinitiators with similar chromophores, are considered to show similar reactivity, as validated by the same polymerization rates described before. As depicted in Figure S2, HMPP has only one weak α C–C bond adjacent to C=O. This bond will undergo Norrish I type cleavage upon photolysis and produce a benzoyl radical and a 2-hydroxy-isopropyl radical. The benzoyl radical has higher activity when adding onto monomers than its counterpart radical. Irgacure 127 has two weak α C–C bonds as cleavage sites; after photolysis, 127 will produce a bis-4,4′-benzoyl methene biradical *via* the same cleavage as HMPP. We originally inferred that if the reaction followed the FRA mechanism, the polymerization rate would increase with increasing 127 components because the bis-4,4′-benzoyl methene biradical would act as a cross-linking agent as divinylbenzene does in the synthesis of ion exchange resins. Figure 4 shows the conversion versus time curves of FT-IR, where the polymerization reactivities are represented based on the SAE monomer with four different photoinitiators. When the SAE monomer was induced by HMPP, at 6.4 wt % concentration, the reaction required about 1800 s to reach 46% conversion, as shown at Figure 4a. As the HMPP concentration increased, the final conversion rate increased and then decreased, indicating an optimal HMPP concentration of approximately 6.4 wt %. Figure 4b shows that increasing the 127 photoinitiator concentration somewhat increased the polymerization conversion. However, the reaction did not display a higher efficiency as we expected, although the final conversion could reach 45% by increasing the concentration to 15.2%. However, the final conversion using 127 was still lower than that using HMPP. These unusual results seemed not to support the FRA mechanism because 127 did not exert a powerful cross-linking effect.

To determine if the SAE would act as a hydrogen donor, we used two hydrogen abstraction photoinitiators (ITX and DETX) reacted with the SAE monomer. Usually the TX series are used in photopolymerization with amine compounds as co-initiators. Unexpectedly, as shown in Figure 4c,d, both ITX and DETX final conversions were higher than that of HMPP, reaching ca. 70%. Hydrogen abstraction photoinitiators rarely produce such a high conversion without amine-like hydrogen donors. This clearly indicated that the SAE monomer was the hydrogen donor, analogous to amines and in agreement with our assumption. Considering this result with the results of quantum chemistry, it is conceivable that the photopolymerization of SAE proceeds by the RMC mechanism.

## 3 Conclusions

Based on the multi-allyl ether monomer, RMC polymerization *via* a stepwise mechanism was applied induced by irradiation. This work also corrects the misconception that polymerization of allyl ether monomers proceeds *via* FRA, including degradative chain transfer. The mechanism of allyl ether monomers may involve three steps (first a HAT reaction, then forming a five-membered cyclopentane reaction, and finally a HAT reaction again). The theoretical results combined with the experimental results can provide a guide for HAT reactions in many monomer functionalizations of the saturated C–H, which played an important role in synthesis fields. This study will supply important findings to activate the alert monomers to polymerize.

## 4 Methods

### 4.1 Computational Methods

**Scheme 2:**
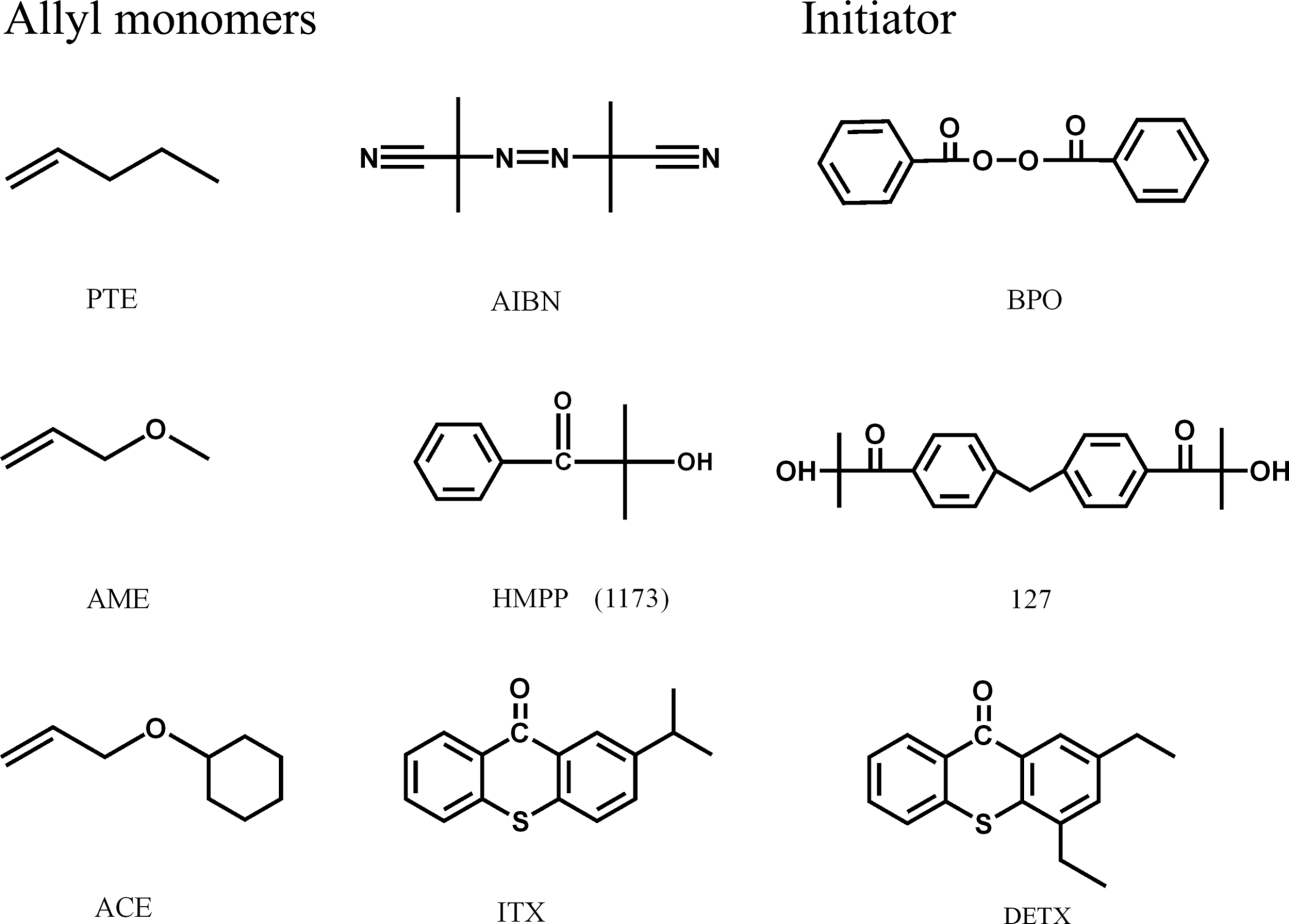
Allyl Monomers and Initiators for the Study

The reactions were based on density functional theory using allyl methyl ether (AME) as a model compound. For simplicity in the subsequent processes, we used the free energy to obtain the value of the thermodynamic properties, such as drive force (Δ*_r_G*) and activation energies (*E*_a_). The majority of geometries were optimized with the functional B3LYP^29,30^ level in conjunction with the 6-311++G(d,p)^31^ basis set, using Gaussian 16^32^ in the gas phase and aqueous solution. The solvation model based on the density^33^ model was used for RMC aqueous solution. To calculate the BDE^34,35^ of C–H bonds, the value derived at the accurate B3LYP/def2tzvp level as well as M06-2X/def2tzvp level was used.^36^ Frequency calculations were performed on the optimized geometries at the same level to ensure that the systems were in local minima without imaginary vibrational frequencies and extract the thermal energy contributions at 298.15 K and 1 atm. For each optimized transition state, the intrinsic reaction coordinate calculation has been performed to find the geometry of reactants and products associated with the transition state in the distortion/interaction model. The wavefunction used in our analyses was produced under the same level in order to obtain the ESP, which used Multiwfn 3.6^37^ and VMD 1.9.^38^ The rate constants (*k*) and tunneling factors (κ_(*T*)_) are obtained by KiSThelP.^39^ The detailed coordinates of compounds are shown in the Supporting Information. Thermal initiators include 2,2′-azobis(2-methylpropionitrile) (AIBN) and benzoyl peroxide (BPO); photoinitiators include 2-isopropylthioxanthone (ITX), 2-hydroxy-2-methyl-1-phenyl-1-propanone (HMPP), 2,4-diethylthioxanthone (DETX), and 2-hydroxy-1-{4-[4-(2-hydroxy-2-methyl-propionyl)-benzyl]-phenyl}-2-methylpropan-1-one (Irgacure 127) (Scheme 2). Pentene (PTE), allyl methyl ether (AME), and allyl cyclohexyl ether (ACE) are all allyl monomers only with a different substituted group.

### 4.2 Experimental Section

The RT-IR spectra of SAE were recorded on a modified Bruker Tensor II spectrophotometer.^40^ The profiles of photopolymerization were recorded by real-time FT-IR.^41,42^ FT-IR (KBr, cm^–1^): 3383 (υ O–H), 2923 (υ C–H), 1647 (υ C=C), 1423 (υ C–C), 1068 (υ C-O-C), 929 (υ CH=CH). For comparison with HMPP, three commercial photoinitiators of low molecular weight, namely, 127, ITX, and DETX, were chosen for FT-IR use. It is designed to allow LED light to irradiate a horizontal sample of SAE using a fiber-optic cable and MCT detector. The light source used for irradiation was a Xenon lamp HAMAMATSU L9566 (28 mW**·**cm^–2^). The rate of reaction was monitored at 1647 cm^–1^, a carbon–carbon double bond absorption peak. The conversion was calculated from
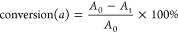
3where *A*_0_ and *A*_t_ are the *cis* H-C=CH_2_ peak areas before and after exposure (at a given time *t*). The details of materials and the synthesis procedure of SAE are shown in the Supporting Information.

## Supporting Information Available

(Figure S1) Mechanistic study of SAE polymerization (page S3); (Figure S2) photolysis reaction of cleavage and hydrogen abstraction photoinitators (page S4); experimental data and materials (page S2); synthesis of monomer SAE (page S2); references (page S5); and main data from the computational part (pages S4–S21) (PDF)
