# Supplementary material for: Density Functional Theory Guide for an Allyl Monomer Polymerization Mechanism: Photoinduced Radical-Mediated [3 + 2] Cyclization
Source: ACS Omega. 2021 Jun 8;6(24):15608–16. doi: 10.1021/acsomega.1c00165 (PMC8223207; doi:10.1021/acsomega.1c00165)
Supplement: Supplementary file 1 — ao1c00165_si_001.pdf [file ao1c00165_si_001.pdf]

Supporting Information

DFT-Guided for Allyl Monomers Polymerization Mechanism: Photoinduced Radical-Mediated [3+2] Cyclization

Xiaotian Zhao [#] [a], Wanqiu Huang [#] [c], Shibo Lin [a], Xi Chen [a], Xirui Guo [a], Dehong Zou [a], Guodong Ye* [b]

[a] *Chengdu Second Peoples Hospital, Chengdu 610017, P.R. China*

[b] *The Fifth Affiliated Hospital of Guangzhou Medical University, Guangzhou 510799, P.R. China*

[c] *Guangdong Pharmaceutical University, Guangzhou 510006, P.R. China*

[#] These authors contributed to the work equally and should be regarded as co-first authors

***Correspondence**:

Guodong Ye

Tel: +86-13229494539

email: gzhygd@gzhmu.edu.cn

Keywords: Allyl ether, Photopolymerization, DFT, Radical-mediated cyclization reaction, FRA, HAT

Content

EXPERIMENTAI DATA 2

THE MAIN DATA FROM COMPUTATIONAL PART 6

EXPERIMENTAI DATA

1. Materials

Sucrose, allyl bromide, sodium hydroxide, and tetrabutylammonium bromide were all reagent grade and were purchased from Aladdin Reagent Co., Ltd. (Shanghai, China). The 2-hydroxy-2-methyl-1-phenyl-1-propanone (HMPP, Irgacure 1173), 1,1′-(methylene-di-4,1-phenylene)bis[2-hydroxy-2-methyl-1-propanone] (Irgacure 127), isopropylthioxanthone (ITX), and 2,4-diethyl-9H-thioxanthen-9-one (DETX) were supplied by TCI Development Co., Ltd. (Shanghai, China).

2. Synthesis of monomer SAE

SAE monomer was synthesized via a modified procedure previously reported by Ortiz [1,2]. Allyl bromide (5.04 mL, 5.84×10^{-2} mol) was added dropwise to a solution of sucrose (5 g, 0.0146 mol) and powdered sodium hydroxide (3.5 g, 0.8764 mol) in water (20 mL) and stirred for 20 min. Then tetrabutylammonium bromide (0.094 g, 2.8×10^{-4} mol) was added as a phase transfer catalyst. The reaction mixture was heated to 70 °C and stirred for 24 h. After this time, the reaction mixture was extracted with four 30 mL portions of ethyl acetate and dried over magnesium sulfate. The solvent was concentrated under reduced pressure before being purified by column chromatography, using silica gel as stationary phase and ethyl acetate as mobile phase. A viscous liquid was obtained with yields of 64%. H-NMR (400 MHz, $CDCl_3$, δ ppm): 3.13-3.82 (m, 14H, C-H cycle, O-H), 3.85-4.41(m, 5H, CH_2-O), 5.20 (m, 8H, -CH_2=CH_2 on the ethylene group), 5.86 (m, 2H, -CH_2=CH_2 on the ethylene group).

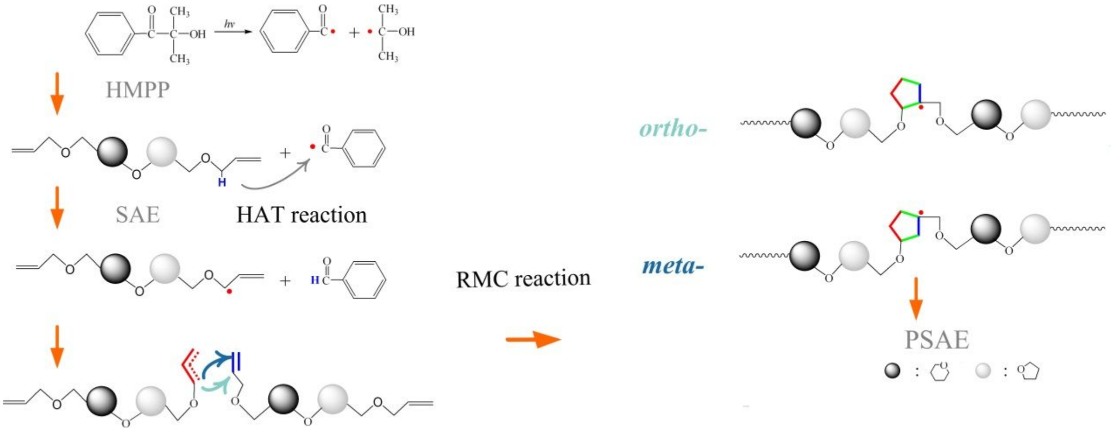

Figure S1 Mechanistic study of SAE polymerization

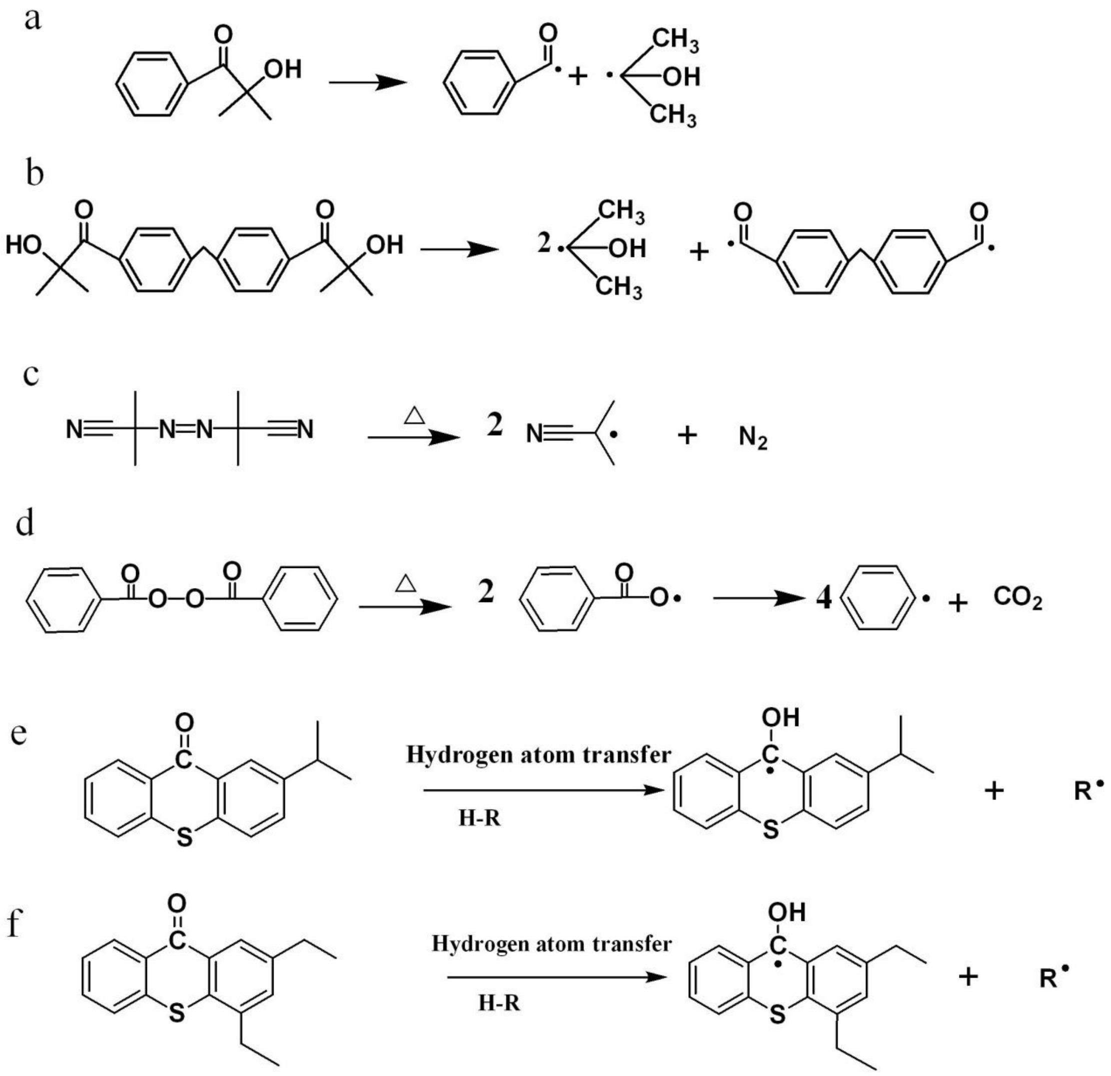

Figure S2 Photolysis reaction of cleavage and hydrogen abstraction photoinitators

References

(1) Acosta Ortiz, R.; Martinez, A.; García Valdez, A; Berlanga Duarte, M. Preparation of a Crosslinked Sucrose Polymer by Thiol–Ene Photopolymerization using Dithiothreitol as Comonomer. *Carbohyd. Polym.* **2010**, *82*, 822–828.

(2) Ortiz, R; Garcia Valdéz, A; Martinez Aguilar, M; Berlanga Duarte, M. An Effective Method to Prepare Sucrose Polymers by Thiol-Ene Photopolymerization. *Carbohyd. Polym.* **2009**, *78*, 282–286.

THE MAIN DATA FROM COMPUTATIONAL PART

xyz coordinates for B3LYP/6-311++g(d,p) optimized geometries

HMPP+benzoyl TS in HAT Reaction

	X	Y	Z
C	-2.679127	-0.278465	-1.415501
C	-2.815468	-1.600799	-1.586403
C	-2.727623	0.384058	-2.276712
H	-2.781591	-2.284549	-0.746871
H	-2.983784	-2.021289	-2.570449
C	-2.404997	0.400905	-0.149447
H	-2.950732	1.344062	-0.016049
O	-2.472911	-0.432771	0.964280
C	-2.418382	0.251440	2.207392
H	-3.246873	0.964855	2.299663
H	-2.504832	-0.503096	2.988774
H	-1.470388	0.789374	2.328036
C	3.673009	0.032104	-0.095702
C	2.627611	0.930730	-0.261368
C	1.301700	0.492099	-0.119175
C	1.033793	-0.846789	0.188736
C	2.086813	-1.743365	0.353971
C	3.402324	-1.304744	0.211599
H	4.698796	0.365814	-0.205208
H	2.814430	1.971271	-0.500266
H	0.006747	-1.176489	0.297429
H	1.882530	-2.780967	0.591384
H	4.221208	-2.004087	0.339512
C	0.176493	1.442294	-0.289385
O	0.258935	2.611762	-0.536824
H	-1.148687	0.866653	-0.217207

HMPP+benzoyl TS in FRA Reaction

	X	Y	Z
C	3.737828	0.820568	0.651235
C	2.466881	1.209616	0.247866
C	1.549736	0.248648	-0.207131
C	1.921307	-1.098713	-0.253006
C	3.196477	-1.484745	0.152862
C	4.103208	-0.527062	0.604196
H	4.446152	1.562673	1.002452
H	2.164812	2.249938	0.276444
H	1.214635	-1.839398	-0.603859
H	3.482254	-2.529678	0.117657
H	5.095739	-0.828761	0.919944
C	0.196282	0.685577	-0.646433
O	-0.218138	1.800086	-0.652300
C	-1.293897	-0.912682	-1.212125
H	-1.148380	-1.402502	-0.256793
H	-0.702149	-1.275109	-2.045054
C	-2.473900	-0.273720	-1.458588
H	-2.700069	0.087267	-2.457787
C	-3.439329	0.146160	-0.399668
H	-3.374925	1.241007	-0.258159
H	-4.473012	-0.067153	-0.722812
O	-3.166505	-0.508843	0.825691
C	-4.007048	-0.080465	1.877706
H	-3.719809	-0.639684	2.768673
H	-3.888456	0.994178	2.075105
H	-5.064915	-0.281819	1.655580

AIBN+ 2-cyano-2-propyl TS in HAT Reaction

	X	Y	Z
H	-0.038394	0.126054	-0.118189
C	-2.202930	0.601095	0.223107
C	-2.751047	1.569443	-0.522853
H	-2.431342	0.534442	1.283932
H	-2.553431	1.654577	-1.586909
H	-3.419256	2.303602	-0.089526
C	-1.248323	-0.396662	-0.269661
H	-1.270584	-0.565970	-1.353367
O	-1.283378	-1.565878	0.474625
C	-0.607653	-2.678997	-0.110248
H	0.462860	-2.486990	-0.222431
H	-0.758170	-3.519716	0.565447
H	-1.038552	-2.919471	-1.089592
C	1.240386	0.729356	0.135359
C	1.188950	2.001368	-0.696898
H	1.144239	1.782204	-1.765746
H	0.311140	2.592265	-0.425000
H	2.078117	2.618702	-0.519554
C	1.201826	0.920749	1.642786
H	0.321991	1.506459	1.922403
H	1.162627	-0.034663	2.169001
H	2.088622	1.463858	1.990943
C	2.176495	-0.246831	-0.325046
N	2.888707	-1.075064	-0.716811

AIBN+ 2-cyano-2-propyl TS in FRA Reaction

	X	Y	Z
C	1.594121	0.037922	0.401744
C	2.440541	-0.806985	-0.368347
C	0.162046	0.524510	-1.157884
H	-0.069618	-0.513750	-1.359820
H	0.941185	0.964866	-1.768843
C	-2.132819	0.869273	-0.107435
H	-2.215923	1.166546	0.955226
H	-2.963052	1.380245	-0.627527
O	-2.266431	-0.530419	-0.230506
C	-3.506244	-1.014139	0.251150
H	-3.506616	-2.094624	0.108283
H	-3.636245	-0.793121	1.319821
H	-4.348649	-0.578781	-0.303700
N	3.101844	-1.485477	-1.039287
C	2.188589	1.377498	0.778553
H	1.405450	2.048313	1.140445
H	2.925003	1.258160	1.583454
H	2.693746	1.851447	-0.065226
C	0.780849	-0.658384	1.470168
H	1.429515	-0.973400	2.297890
H	0.031068	0.024103	1.875983
H	0.270233	-1.540226	1.082503
C	-0.834742	1.340939	-0.678522
H	-0.691601	2.418114	-0.656001

BPO + Phenyl TS in HAT Reaction

	X	Y	Z
C	-2.406322	1.146247	0.850911
C	-3.225943	1.827319	0.048932
H	-2.044234	1.597763	1.771636
H	-3.597782	1.395521	-0.872237
H	-3.549179	2.829970	0.302890
O	-2.426960	-0.791034	-0.556257
C	-2.009596	-2.128094	-0.780727
H	-2.295821	-2.777456	0.057479
H	-2.511491	-2.469408	-1.685763
H	-0.923820	-2.185218	-0.923197
C	3.151070	-0.555392	0.811576
C	1.777542	-0.707730	1.039706
C	0.901863	-0.024976	0.216340
C	1.312708	0.797855	-0.815544
C	2.688168	0.941889	-1.034377
C	3.600208	0.266545	-0.222291
H	3.864351	-1.077713	1.441017
H	1.423843	-1.346224	1.843349
H	0.596641	1.321926	-1.440279
H	3.043014	1.580878	-1.836538
H	4.664362	0.381849	-0.395321
C	-1.885055	-0.216699	0.593691
H	-0.684935	-0.132197	0.465783
H	-1.997128	-0.873212	1.471939

BPO + Benzoate TS in FRA Reaction

	X	Y	Z
C	3.346915	0.178957	-0.051328
H	4.348228	0.024608	-0.491569
H	3.519478	0.567209	0.969689
O	2.632007	-1.034536	-0.004542
C	3.306913	-2.046606	0.725206
H	3.461399	-1.749360	1.770727
H	2.677978	-2.935849	0.695328
H	4.280105	-2.280783	0.273783
C	-1.499029	-2.069583	0.063998
C	-0.863203	-0.852793	0.303488
C	-1.573341	0.345278	0.171626
C	-2.924197	0.310319	-0.194793
C	-3.549497	-0.905010	-0.454339
C	-2.838137	-2.098053	-0.322656
H	-0.948397	-2.996477	0.179823
H	0.177882	-0.835496	0.601614
H	-3.471575	1.242413	-0.267177
H	-4.592321	-0.923651	-0.750293
H	-3.327535	-3.046273	-0.515770
C	-0.962347	1.676441	0.490143
O	-1.627932	2.570072	0.997972
C	2.622857	1.214765	-0.833940
H	3.149986	2.157886	-0.945356
O	0.313525	1.915557	0.272875
C	1.331854	1.105951	-1.294766
H	0.848289	0.140989	-1.311884
H	0.942961	1.868046	-1.955181

Path 1 TS in gas phase:

	X	Y	Z
C	0.335381	-0.401709	1.098407
C	0.397215	-1.783435	1.216663
C	-0.264222	0.129252	1.836015
H	1.157729	-2.349314	0.693260
H	-0.384473	-2.337405	1.720489
C	1.565258	0.372769	0.674021
H	1.291856	1.396018	0.384955
O	2.198870	-0.287903	-0.412726
C	3.382408	0.359689	-0.834108
H	4.128791	0.397033	-0.027868
H	3.787902	-0.216594	-1.666455
H	3.181077	1.385777	-1.173012
H	2.265401	0.440332	1.522692
C	-2.155798	-0.650359	-0.381075
C	-2.285206	-1.884244	-0.899226
H	-2.976290	-0.246728	0.204791
H	-1.494842	-2.341928	-1.484435
H	-3.184769	-2.468022	-0.745467
C	-0.941413	0.136471	-0.477017
H	-0.232586	-0.146374	-1.246204
O	-0.991008	1.515275	-0.407225
C	-1.996549	2.123152	0.393449
H	-2.012412	1.711222	1.408830
H	-1.745026	3.182170	0.441424
H	-2.988816	2.011586	-0.056088

Path 1 TS in water solution:

	X	Y	Z
C	0.221375	-0.714854	0.942728
C	0.143691	-2.087686	0.744683
H	-0.365520	-0.300255	1.759046
H	0.858910	-2.607201	0.117548
H	-0.717046	-2.649890	1.085141
C	1.523461	0.018044	0.737522
H	1.357079	1.099710	0.699837
O	2.154914	-0.411209	-0.468185
C	3.401066	0.244703	-0.675709
H	4.099740	0.029066	0.141925
H	3.814650	-0.131436	-1.611828
H	3.265160	1.330633	-0.749768
H	2.192394	-0.183086	1.587285
C	-2.325789	-0.241888	-0.228623
C	-2.848236	-1.381661	-0.713087
H	-2.901924	0.346143	0.478739
H	-2.301381	-2.003458	-1.415025
H	-3.832580	-1.721563	-0.412015
C	-0.976428	0.199035	-0.526137
H	-0.492760	-0.246412	-1.387050
O	-0.661934	1.546484	-0.487788
C	-1.260086	2.343276	0.544459
H	-1.145989	1.870238	1.522612
H	-0.729279	3.293953	0.535484
H	-2.319618	2.517544	0.340838

Path 2 TS in gas phase:

	X	Y	Z
C	-1.818281	1.463838	-0.308078
C	-1.055011	2.471693	-0.765416
H	-2.806866	1.666926	0.097011
H	-0.063059	2.295549	-1.167436
H	-1.406766	3.495862	-0.739977
C	-1.401490	0.081049	-0.253802
H	-0.588240	-0.236457	-0.901983
O	-2.441612	-0.809552	-0.133041
C	-2.145719	-2.153562	-0.488114
H	-1.377267	-2.582217	0.164480
H	-3.069867	-2.718254	-0.371481
H	-1.808033	-2.216457	-1.529030
C	0.923547	0.476748	1.462385
C	-0.330624	-0.091660	1.567350
H	1.046567	1.540516	1.640102
H	-0.404806	-1.176002	1.575367
H	-1.098994	0.416552	2.138819
C	2.089400	-0.246544	0.879586
H	3.024776	0.056355	1.373986
O	2.193668	0.051666	-0.525203
C	3.341829	-0.523421	-1.119371
H	3.331680	-1.620470	-1.041243
H	3.337114	-0.243105	-2.173415
H	4.265567	-0.150166	-0.654568
H	1.979837	-1.334380	1.008078

Path 2 TS in water solution:

	X	Y	Z
C	-1.699867	1.563245	-0.198493
C	-0.838430	2.510334	-0.610929
H	-2.635101	1.847792	0.278264
H	0.104887	2.252995	-1.081572
H	-1.058766	3.563420	-0.481214
C	-1.436686	0.145867	-0.265558
H	-0.682456	-0.210022	-0.961676
O	-2.567490	-0.645930	-0.190657
C	-2.385716	-1.997205	-0.634750
H	-1.647310	-2.517127	-0.018940
H	-3.352292	-2.488746	-0.537123
H	-2.065478	-2.012103	-1.680573
C	0.890775	0.245541	1.455679
C	-0.393081	-0.265481	1.506399
H	1.079866	1.270770	1.760885
H	-0.520695	-1.338042	1.391259
H	-1.130697	0.218320	2.138125
C	2.016153	-0.483023	0.812038
H	2.913015	-0.473032	1.448076
O	2.356324	0.148313	-0.444410
C	3.462150	-0.493454	-1.073957
H	3.232929	-1.542826	-1.297138
H	3.659885	0.037517	-2.005740
H	4.354818	-0.453533	-0.437481
H	1.744940	-1.529382	0.622808

Path 3 TS in gas phase:

	X	Y	Z
C	0.949842	1.802791	-0.215909
C	-0.147314	2.421541	0.460838
H	1.285705	2.220622	-1.161565
H	-0.322621	2.149486	1.495867
H	-0.336707	3.466788	0.239679
C	1.556251	0.671645	0.216486
H	1.267312	0.172952	1.138346
O	2.530461	0.067245	-0.513730
C	3.240967	-0.970743	0.153991
H	2.555418	-1.742384	0.521194
H	3.917389	-1.406604	-0.579802
H	3.822689	-0.571104	0.991982
C	-2.330192	0.487386	0.197522
C	-2.007017	1.731777	-0.303688
H	-2.834655	0.406247	1.156501
H	-1.691629	1.817625	-1.338824
H	-2.539286	2.595469	0.081390
C	-1.933868	-0.798617	-0.439988
H	-2.829386	-1.374811	-0.732338
O	-1.173123	-1.592610	0.479263
C	-0.891130	-2.882777	-0.025717
H	-0.283537	-2.836045	-0.941168
H	-0.334200	-3.418792	0.744207
H	-1.813166	-3.438979	-0.248723
H	-1.347263	-0.615566	-1.352220

Path 3 TS in water solution:

	X	Y	Z
C	-0.137284	1.901268	-0.382375
C	-1.384403	2.113572	0.282544
H	-0.058936	2.150435	-1.437710
H	-1.402978	2.025090	1.363806
H	-2.050613	2.867981	-0.121715
C	0.917258	1.293450	0.209684
H	0.917700	1.005920	1.256779
O	2.042768	0.971295	-0.496485
C	3.122249	0.451918	0.295728
H	2.792000	-0.415476	0.872495
H	3.902869	0.153878	-0.401817
H	3.504244	1.222918	0.970610
C	-2.189318	-0.682243	0.345849
C	-2.682473	0.500368	-0.165618
H	-2.447488	-0.989822	1.355658
H	-2.602383	0.684960	-1.232338
H	-3.539623	0.960597	0.315247
C	-1.197556	-1.524585	-0.376343
H	-1.643083	-2.472877	-0.718116
O	-0.097144	-1.843863	0.495625
C	0.930470	-2.553391	-0.189355
H	1.351090	-1.947395	-1.000752
H	1.712545	-2.777308	0.536334
H	0.547597	-3.492168	-0.609037
H	-0.821590	-0.998337	-1.263244

Path 4 TS in gas phase:

	X	Y	Z
C	1.207550	0.183234	-1.045189
C	0.334617	1.308966	-1.177282
H	0.978280	-0.728404	-1.590275
H	0.696500	2.268046	-0.823508
H	-0.255745	1.371719	-2.085754
C	2.277450	0.156769	-0.215471
H	2.583961	1.023592	0.366725
O	3.017494	-0.970140	-0.047155
C	4.238782	-0.804567	0.666180
H	4.059598	-0.373330	1.657663
H	4.670046	-1.798000	0.778501
H	4.934782	-0.166559	0.110586
C	-1.343647	1.206800	0.130703
C	-0.925962	1.503131	1.413175
H	-1.838505	2.000672	-0.423663
H	-0.616666	0.720839	2.097910
H	-0.810185	2.527456	1.748110
C	-1.834722	-0.182954	-0.192896
H	-1.825972	-0.363710	-1.277677
O	-3.166597	-0.330145	0.293074
O	-3.716858	-1.597041	-0.006409
H	-3.134306	-2.409226	0.452194
H	-4.728879	-1.613725	0.399591
H	-3.764048	-1.771791	-1.091419
H	-1.179297	-0.931333	0.276811

Path 4 TS in water solution:

	X	Y	Z
C	1.160901	0.264285	-1.090540
C	0.309403	1.412042	-1.134719
H	0.902308	-0.605765	-1.687806
H	0.693946	2.339513	-0.725737
H	-0.322683	1.533573	-2.007715
C	2.221195	0.173370	-0.254102
H	2.537952	0.992826	0.385319
O	2.956038	-0.974812	-0.160532
C	4.120157	-0.880620	0.676568
H	3.836607	-0.599459	1.694334
H	4.577064	-1.868462	0.681563
H	4.824221	-0.149402	0.270664
C	-1.283067	1.206512	0.239772
C	-0.764974	1.317329	1.516419
H	-1.779181	2.078256	-0.179597
H	-0.408710	0.442040	2.050605
H	-0.575192	2.285308	1.966606
C	-1.817306	-0.120086	-0.230127
H	-1.923246	-0.145102	-1.320483
O	-3.109553	-0.345315	0.361406
C	-3.687982	-1.566786	-0.090251
H	-3.054629	-2.422547	0.174066
H	-4.656017	-1.670168	0.400967
H	-3.832811	-1.555236	-1.177549
H	-1.141478	-0.930644	0.069651

AME+*Ortho*- TS

	X	Y	Z
C	1.423588	0.688803	-0.082727
C	1.438078	1.498830	1.224566
H	0.806519	1.208657	-0.825965
H	2.244225	1.131991	1.864986
H	1.612165	2.564052	1.052066
C	2.781930	0.446155	-0.722844
H	2.656341	-0.165429	-1.627872
O	3.639683	-0.219907	0.195638
C	4.909882	-0.512252	-0.348563
H	5.440955	0.403484	-0.646164
H	5.486543	-1.020314	0.425427
H	4.830338	-1.169875	-1.225905
H	3.234794	1.404108	-1.023785
C	-0.389754	-0.122195	1.296682
C	0.062484	1.214910	1.877768
H	-0.769461	-0.863476	1.998098
H	0.122366	1.197622	2.970116
H	-0.649800	2.011735	1.624874
C	0.664384	-0.592063	0.299420
H	1.360326	-1.288967	0.795326
O	0.175718	-1.220969	-0.884328
C	-0.303406	-2.538642	-0.685025
H	-1.209886	-2.555047	-0.068130
H	-0.546904	-2.942423	-1.668648
H	0.462576	-3.173313	-0.217055
H	-3.155636	1.220631	0.582776
C	-2.349968	1.021062	-1.413383
C	-2.263987	2.324120	-1.704530
H	-2.099647	0.276016	-2.163001
H	-2.508199	3.091365	-0.975799
H	-1.944098	2.663738	-2.682554
C	-2.695557	0.484695	-0.090612
H	-1.587282	0.180973	0.515491
O	-3.448583	-0.693374	-0.184587
C	-3.992295	-1.133759	1.047512
H	-4.653170	-0.372211	1.482836
H	-4.573058	-2.031498	0.835736
H	-3.206839	-1.375803	1.773782

AME+*Meta*- TS

	X	Y	Z
C	0.805661	-0.187949	1.035345
C	-0.022573	0.911316	1.675438
H	1.284554	-0.917020	1.685787
H	-1.053131	0.621543	1.900161
H	0.429362	1.216788	2.628961
C	1.726563	0.417242	-0.009230
H	1.507479	0.017190	-1.012653
O	3.081573	0.082747	0.301750
C	3.998643	0.381739	-0.734606
H	4.073558	1.461164	-0.922629
H	4.973834	0.013775	-0.413567
H	3.718493	-0.118798	-1.672547
C	0.038504	2.089314	0.675833
C	1.437628	1.942603	0.041233
H	-0.061756	3.046929	1.194418
H	1.515520	2.429812	-0.935081
H	2.185480	2.396185	0.698950
C	-1.063607	2.033402	-0.379440
H	-0.925641	2.854233	-1.100506
O	-2.326875	2.160167	0.255896
C	-3.408145	2.171726	-0.654139
H	-3.458327	1.239066	-1.234265
H	-4.323006	2.275318	-0.069731
H	-3.334725	3.014815	-1.355770
H	-1.023146	1.089721	-0.946186
H	-1.827552	-1.591296	-0.204945
C	-0.299380	-2.259744	-1.584047
C	-0.786854	-1.708674	-2.703238
H	0.571275	-2.909579	-1.634955
H	-1.666074	-1.071893	-2.691960
H	-0.321270	-1.880707	-3.666336
C	-0.817677	-2.022106	-0.231285
H	-0.082969	-1.104813	0.318850
O	-0.700772	-3.163650	0.570616
C	-1.310307	-3.042048	1.845367
H	-2.384794	-2.835558	1.750919
H	-1.172210	-3.995953	2.353878
H	-0.847100	-2.245960	2.439728

xyz coordinates for B3LYP/def2tzvp optimized geometries

	PTE				AME				ACE		
	X	Y	Z		X	Y	Z		X	Y	Z
C	-1.414615	0.336749	0.270917	C	1.324400	-0.382700	0.200562	C	3.349810	-0.135255	-0.385844
C	-2.480307	-0.211050	-0.300280	C	2.432487	0.191114	-0.247508	C	4.460713	0.244580	0.230300
H	-1.492179	1.349804	0.660821	H	1.328909	-1.429032	0.491142	H	3.342201	-0.273064	-1.462994
H	-2.453365	-1.217041	-0.705169	H	2.450174	1.230818	-0.555197	H	4.491079	0.401519	1.302901
H	-3.419236	0.321891	-0.382283	H	3.364456	-0.354625	-0.320914	H	5.382595	0.410279	-0.312468
C	-0.075139	-0.317917	0.422484	H	0.016412	0.319897	0.367660	O	1.024435	0.316260	-0.334571
H	0.168967	-0.394146	1.489543	H	-0.222025	0.415957	1.439475	C	-0.282456	0.128479	0.206232
H	-0.120303	-1.341745	0.038877	H	0.067319	1.335516	-0.049286	C	-1.121141	1.329923	-0.216372
C	2.425997	-0.193662	-0.076016	O	-1.000563	-0.432784	-0.273055	C	-0.924959	-1.181369	-0.260302
C	2.446446	-1.214140	-0.466633	C	-2.286472	0.120082	-0.096156	H	-0.210696	0.116993	1.305544
H	3.208779	0.371646	-0.585452	H	-2.353526	1.130075	-0.521580	C	-2.568573	1.213293	0.271784
H	2.686876	-0.241213	0.984281	H	-2.992114	-0.528912	-0.612826	H	-1.098729	1.391922	-1.309443
C	1.055548	0.448288	-0.279688	H	-2.559647	0.172113	0.966281	H	-0.654482	2.242275	0.161256
H	1.074422	1.478689	0.089856					C	-2.370082	-1.305676	0.236827
H	0.830694	0.511808	-1.348346					H	-0.904391	-1.195166	-1.355176
								H	-0.338838	-2.038292	0.080257
								C	-3.214394	-0.099068	-0.180904
								H	-3.149380	2.066865	-0.086583
								H	-2.588525	1.265208	1.366560
								H	-2.813617	-2.230605	-0.140471
								H	-2.372658	-1.386794	1.330060
								H	-4.224556	-0.184593	0.228313
								H	-3.318828	-0.092907	-1.271850
								C	2.054549	-0.422880	0.302935
								H	1.845095	-1.500710	0.245987
								H	2.117441	-0.155182	1.366743
